# Identification of Distinctive Patterns of USP19-Mediated Growth Regulation in Normal and Malignant Cells

**DOI:** 10.1371/journal.pone.0015936

**Published:** 2011-01-17

**Authors:** Yu Lu, Nathalie Bedard, Simone Chevalier, Simon S. Wing

**Affiliations:** 1 Polypeptide Laboratory, Department of Medicine, McGill University Health Centre Research Institute, McGill University, Montreal, Quebec, Canada; 2 Division of Urology, Department of Surgery, McGill University Health Centre Research Institute, McGill University, Montreal, Quebec, Canada; Texas A&M University, United States of America

## Abstract

We previously reported that the USP19 deubiquitinating enzyme positively regulates proliferation in fibroblasts by stabilizing KPC1, a ubiquitin ligase for p27^Kip1^. To explore whether this role of USP19 extends to other cellular systems, we tested the effects of silencing of USP19 in several human prostate and breast models, including carcinoma cell lines. Depletion of USP19 inhibited proliferation in prostate cancer DU145, PC-3 and 22RV1 cells, which was similar to the pattern established in fibroblasts in that it was due to decreased progression from G1 to S phase and associated with a stabilization of the cyclin-dependent kinase inhibitor p27^Kip1^. However, in contrast to previous findings in fibroblasts, the stabilization of p27^Kip1^ upon USP19 depletion was not associated with changes in the levels of the KPC1 ligase. USP19 could also regulate the growth of immortalized MCF10A breast epithelial cells through a similar mechanism. This regulatory pattern was lost, though, in breast cancer MCF7 and MDA-MB-231 cells and in prostate carcinoma LNCaP cells. Of interest, the transformation of fibroblasts through overexpression of an oncogenic form of Ras disrupted the USP19-mediated regulation of cell growth and of levels of p27^Kip1^ and KPC1. Thus, the cell context appears determinant for the ability of USP19 to regulate cell proliferation and p27^Kip1^ levels. This may occur through both KPC1 dependent and independent mechanisms. Moreover, a complete loss of USP19 function on cell growth may arise as a result of oncogenic transformation of cells.

## Introduction

Eukaryotic cell cycle progression is dependent on the precisely timed activation and inactivation of a series of cyclin-dependent kinases (CDKs) [1], [2]. Activation and inactivation of CDKs occur by interaction with positive regulatory cyclins and negative regulatory CDK inhibitors (CKIs) respectively. For example progression through G1 and into S phase is controlled by the CDK2 kinase activity and is stimulated by the sequential binding of cyclins D and E. Premature progression is prevented by the several CKIs such as p16, p27^Kip1^ and p21. One of the best studied CKIs is p27^Kip1^, which intervenes mainly at the G1-S transition [3]. Upon binding to the cyclin E-CDK2 complex, p27^Kip1^ inhibits CDK2 activity, thereby inhibiting cell cycle progression and cell proliferation. Silencing of p27^Kip1^ results in enhanced cell proliferation [4]. Consistent with this growth inhibitory role, p27^Kip1^ knockout mice are larger in size than wild-type littermates. They develop multi-organ hyperplasia and exhibit increased susceptibility to cancer [5], [6], [7].

Studies in cancer also support an important role of p27^Kip1^ in regulating cell proliferation. For example, reduced abundance of p27^Kip1^ is often related to high tumor grade and poor prognosis in a variety of human cancers, including prostate and breast cancers [4]. Seven out of nine multivariate analyses of 1,464 prostate cancer patients show that reduced nuclear p27^Kip1^ is an independent predictor of decreased time from prostatectomy to disease recurrence [4]. A systematic review of 18 studies involving 6,216 breast cancer patients showed that reduced levels of p27^Kip1^ were an independent prognostic factor for shortened overall survival and disease-free survival [8]. Such reduction of p27^Kip1^ levels in cancer cells is often attributed to increased degradation mediated by the ubiquitin-proteasome pathway [9]. In this process, a cascade of enzymes (E1: ubiquitin activating enzyme, E2: ubiquitin conjugating enzyme and E3: ubiquitin-protein ligase) builds up polyubiquitin chains on p27^Kip1^. Ubiquitinated p27^Kip1^ is then delivered to the 26S proteasome for degradation [10]. Multiple E3s have been implicated in the ubiquitination of p27^Kip1^. At the transition from G0 to G1, an E3 complex, KPC (Kip1 ubiquitination-promoting complex), targets p27^Kip1^ for degradation in the cytoplasm. KPC contains a Ring-finger catalytic subunit KPC1 that polyubiquitinates p27^Kip1^ and an adaptor protein KPC2 that delivers ubiquitinated p27^Kip1^ to the 26S proteasome for degradation [11], [12], [13]. At the G1-S transition, p27^Kip1^ becomes a substrate of Skp2, which is an F-box protein that functions as the substrate recognition component of a SCF-type Cullin Ring Ligase complex in the nucleus [14], [15]. Recently, Pirh2, a RING finger type E3 expressed both in the nucleus and cytoplasm, has also been found to target p27^Kip1^ for degradation in a panel of tumor cell lines, including T98G (glioblastoma), MCF7 (breast carcinoma), Colo320DM (colon carcinoma), and HeLa cells (uterine cervical carcinoma) [16], [17].

Protein ubiquitination is a reversible process. Ubiquitin can be deconjugated by a family of deubiquitinating enzymes (DUBs) [18], [19]. Although the E3s for polyubiquitination and degradation of p27^Kip1^ have been extensively studied, very little is known about the role of DUBs in regulating p27^Kip1^. We recently identified USP19 as a DUB that indirectly modulates levels of p27^Kip1^ by a novel mechanism [20]. USP19 deubiquitinates and thereby stabilizes the KPC1 ligase for p27^Kip1^, and so indirectly promotes degradation of p27^Kip1^ and subsequent cell proliferation. This mechanism was established in rat FR3T3 fibroblasts and confirmed in mouse embryonic fibroblasts [20]. In the present investigation, we tested whether the ability of USP19 to regulate p27^Kip1^ and cell proliferation applies more broadly. We found that the cell context matters with distinctive patterns of USP19 regulation in a panel of human breast and prostate derived cell lines. We also report that some of the differences may be attributed, at least in part, to oncogenic cell transformation.

## Methods

### Reagents

Cell culture media, and Lipofectamine Plus reagent were from Invitrogen. Propidium iodide, RNase A, cycloheximide and anti-tubulin antibody were from Sigma. Antibodies to p16, p21 and Skp2 were from Santa Cruz Biotechnology, and to p27^Kip1^, Ras, caspase 3 from Cell Signaling, to PARP from Roche and to Pirh2 from Calbiochem. Antibodies to KPC1 were a gift from Dr. K. Nakayama [11]. USP19 antibodies were generated by immunizing rabbits with a fragment containing the first 129 amino acids of USP19 [20].

### Cell culture

DU145, PC-3, 22RV1, LNCaP clone FGC, MCF10A, MCF 7 and MDA-MB-231 were obtained from the American Type Culture Collection (ATCC). Parental FR3T3 cells were a gift from Dr. A. Nepveu. Cells were routinely maintained in culture medium RPMI1640: DU145, PC-3, 22RV1, LNCaP; MEBM medium (Lonza): MCF10A; DMEM (high glucose): MCF 7, MDA-MB-231, and FR3T3, supplemented with 10% fetal bovine serum (FBS) and antibiotic-antimycotic preparations (Invitrogen). Puromycin (2 µg/ml) was included for the culture of Ras-V12 transformed FR3T3 and FR3T3-vector cells. Proliferation was assessed by determining cell number. At the end of the treatment period, cells were harvested from each well using trypsin, stained with trypan blue dye and non-stained cells counted on a hemocytometer. Shown in Figures are mean cell numbers ± SE of triplicate samples from a representative experiment. Each experiment was repeated two to four times.

### siRNA transfection

For typical transfection of small interfering RNA (siRNA) oligonucleotides, 6.5×10^5^ 22RV1 cells, 4×10^5^ DU145 cells, 4.5×10^5^ DU145-Vect cells, 4.5×10^5^ DU145-USP19 cells, 3×10^5^ PC-3 cells, 7×10^5^ LNCaP cells, 2.4×10^5^ HEK293 cells, 5×10^5^ MCF7, 3.8×10^5^ MDA-MB-231 and 1.2×10^6^ MCF10A cells were seeded into 60 mm plates. SiRNAs were used at 50 nM for all cell lines with the exception of 55 nM for MCF10A cells. For LNCaP cells and MDA-MB-231, cell plating and transfection were performed simultaneously using LNCaP Transfection Reagent (Altogen Biosystems) and MDA-MB Transfection Reagent (Altogen Biosystems) respectively. For MCF7, 22RV1, DU145, DU145-Vect, DU145-USP19 and PC-3 cells, transfection took place 24 hours after plating using the trans IT-TKO Transfection Reagent (Mirus) for MCF7, and transfection reagent Lipofectamine Plus (Invitrogen) for the rest of cell lines. For MCF10A cells, transfection was performed 48 hours after plating using X-tremeGENE siRNA Transfection Reagent (Roche). The transfections were otherwise carried out according to the protocols of the manufacturers of the indicated transfection reagent. SiRNA oligonucleotides were purchased from Integrated DNA Technologies (Coralville, IA) and designed against the following USP19 coding sequences:

Human USP19 siRNA, h1: GGAGGAGATGGCAGTGGCA; and h6: GGATGGAGATCCTAGGAAA;

Rat USP19 siRNA, #43: GGCGTGACAAGATCAATGACTTG; and #1: AAAGTGCAGACTCACAAGGGT

The sequence of the nonspecific control oligonucleotide was GUCAGCGUGCAGAUAGAGUUU.

### Cell cycle Analysis

Forty-eight hours after USP19 or control siRNA transfection, DU145 or PC-3 cells were trypsinized, washed and resuspended in 1 ml of ice-cold PBS, subsequently fixed by drop-wise addition of 3 ml ice-cold 70% ethanol and stored in −20°C overnight. FBS (100 µl) was added to the fixed cells which were then centrifuged, washed twice in PBS, and incubated in PBS containing 100 µg/ml RNase A and 10 µg/ml Propidium Iodide for 15 min at 37°C. Stained cells were counted in FACScan (Becton Dickinson), and data were analyzed utilizing WinMDI 2.9 software. For each sample 1×10^4^ cells were recorded.

### BrdU incorporation analysis

Forty-eight hours after USP19 or control siRNA transfection, DU145 or PC-3 cells were incubated in fresh culture medium with 10 µM BrdU (Roche) for 55 min. Cells were then washed 3 times with PBS, trypsinized, and resuspended in 0.5 ml PBS. The cells were stained according to the manufacturer's protocol, and counted in a FACS Calibur analyser. Data were analyzed utilizing WinMDI 2.9 software.

### p27^Kip1^ Northern blot analysis

RNA was isolated from DU145 cells 48 h after USP19 or control siRNA transfection using Trizol reagent (Invitrogen) according to the manufacturer's protocol. RNA (20 µg) was electrophoresed on a 1% agarose gel and transferred to a nylon membrane. The membranes were hybridized with ^32^P labeled p27^Kip1^ probes, and subjected to autoradiography. Single stranded cDNA probes were prepared by PCR based amplification using p27^Kip1^ in pCMV (Addgene) as a template and the p27^Kip1^ reverse primer: GAAGAATCGTCGGTTGCAGGTCGCT.

### Stable cell lines expressing USP19 or Ras

3×10^5^ DU145 cells were transduced with retrovirus encoding pLXSN empty vector (Clontech) or N-terminal 3× Flag-tagged USP19 in the presence of 8 µg/ml polybrene. The stable clones were selected by growing the cells in culture media containing G418 (400 µg/ml). 6×10^5^ FR3T3 cells were transduced with retrovirus carrying pBABE-puro-vector/Ras (G12V) in the presence of 8 µg/ml polybrene. The stable clones were selected by growing the cells in culture media containing puromycin (2 µg/ml).

## Results

### Effects of depletion of USP19 on cell proliferation in prostate cancer cell lines

**Figure 1:**
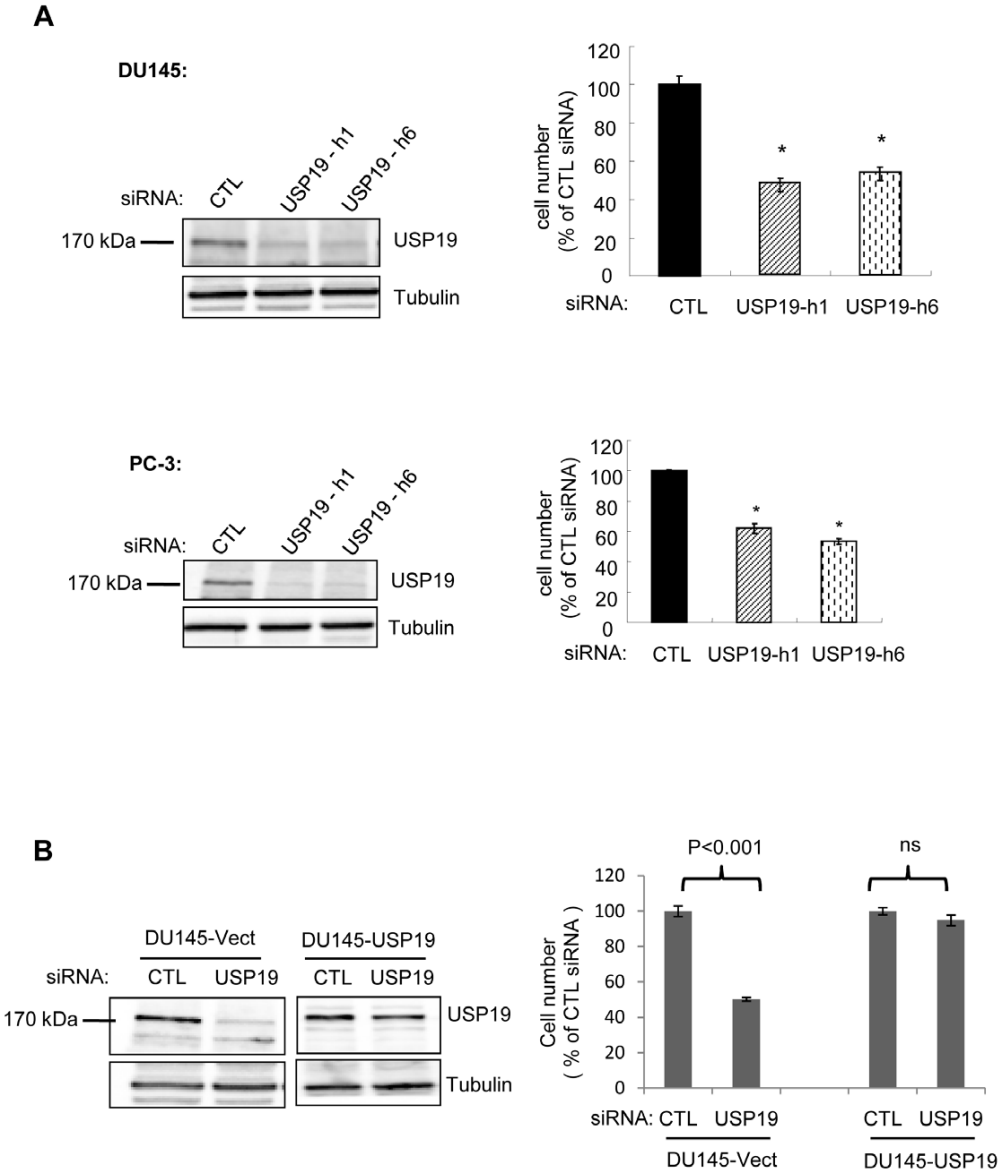
Depletion of USP19 in prostate cancer DU145 and PC-3 cells leads to reduced proliferation. (**A**) DU145 or PC-3 prostate cancer cells were transfected with USP19 siRNA oligonucleotides h1 or h6 or nonspecific control siRNA oligonucleotides (CTL). Forty-eight hours after transfection, cells were harvested and counted. Shown are means ± SE of triplicate samples. *, *P*<0.001 compared to CTL. (**B**) Expressing USP19 in DU145 cells depleted of endogenous USP19 restores normal proliferation. DU145 cells stably expressing empty vector or rat USP19 that is resistant to USP19 siRNA oligonucleotide h1 were transfected with h1 or nonspecific (CTL) siRNA oligonucleotide. After 48 h, cells were harvested and counted. Shown are means ± SE of triplicate samples. ns, not significant. Cell lysates from DU145-Vect (300 µg of protein) or DU145-USP19 (20 µg of protein) were analyzed by immunoblotting with the indicated antibodies.

To investigate the role of USP19 in regulating prostate cancer cell growth, we depleted this enzyme in several cell lines. We transfected USP19 siRNA into androgen-independent DU145 and PC-3 cells as well as androgen-responsive LNCaP and 22RV1 cell lines (Fig. 1, Fig. S1). Forty-eight hours after transfection, the USP19 protein levels were suppressed by ∼75% in all cell lines. Depletion of USP19 resulted in a ∼50% decrease in cell number in DU145 and PC-3 cells compared to that of control cells transfected with a nonspecific siRNA (Fig. 1A). The two independent USP19 siRNAs tested led to similar reductions in cell growth, suggesting that this effect in the USP19 depleted human cells was unlikely to be due to off-target effects. Indeed this was tested by the stable overexpression in DU145 cells of rat USP19 that is resistant to targeting by human USP19 siRNA. Our strategy resulted in a cell line in which USP19 levels did not fall and in which growth was no longer inhibited upon USP19 depletion (Fig. 1B), thereby confirming the ability of rat USP19 to reverse the effect of human USP19 siRNA and thus specificity of action of the siRNA oligonucleotide. The silencing of USP19 was also growth-inhibitory in 22RV1 cells (Fig. S1A), but not in LNCaP cells despite a similar extent of silencing of USP19 (Fig. S1B). These results indicated that USP19 modulates growth in several but not all prostate cancer cell lines. Moreover, their differential growth response does not appear strictly due to their androgen-sensitivity. However, we cannot rule out the possibility that USP19 may mediate some of its effects on growth through androgen receptor dependent pathways.

### Depletion of USP19 results in defects in cell cycle progression

**Figure 2:**
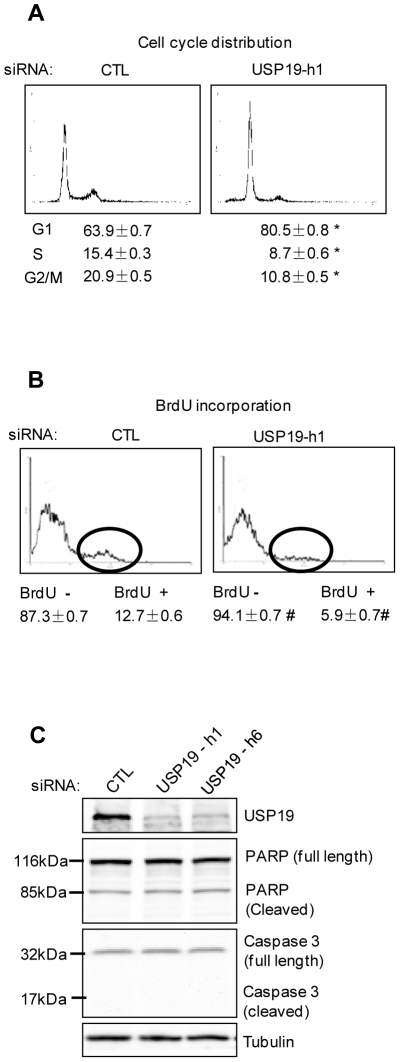
Depletion of USP19 in DU145 cells results in defects in cell cycle progression. (**A**) DU145 cells depleted of USP19 show accumulation in G1 phase. Forty-eight hours after transfection with USP19 siRNA oligonucleotide h1 or control oligonucleotide (CTL), DU145 cells were harvested and subjected to FACS analysis. Shown are representative profiles and distribution of cells in various phases of the cycle (expressed as % of total cells ± SE from three experiments). *, *P*<0.001 compared to CTL. (**B**) DU145 cells depleted of USP19 show decreased entry into S phase. Forty-eight hours after transfection as in (A), cells were incubated with BrdU (10 µM) for 55 min and analyzed by FACS to quantify S phase cells (identified as BrdU positive cells). Shown are representative profiles and quantitation of percentages of cells that were BrdU positive or negative. Circles identify the BrdU positive cells in S phase. #, *P*<0.005 compared to CTL. (**C**) Depletion of USP19 did not affect the levels of poly (ADP-ribose) polymerase (PARP) or activated caspase-3 in DU145 cells. Cells were transfected as in (A) and harvested 48 h later to analyze protein from lysates by immunoblotting with the indicated antibodies.

To determine whether the reduction in the number of prostate cancer cells upon USP19 depletion resulted from a delay in cell cycle progression, we measured the cell cycle distributions of DU145 cells transfected with control or USP19 siRNAs. Depletion of USP19 resulted in a significant increase in the percentage of cells in G0/G1 phase, which was accompanied by a decrease in S and G2/M phases compared to that in control cells (Fig. 2A). Concomitantly, BrdU incorporation, a specific indicator of DNA synthesis in S phase was reduced ∼50% in the cells with USP19 depletion (Fig. 2B). There was no evidence of a sub-G1 peak (Fig. 2A) and no detectable effects of USP19 depletion on the levels of apoptotic markers, such as poly (ADP-ribose) polymerase (PARP) or activated caspase-3 (Fig. 2C). Similar effects on cell cycle distribution and BrdU incorporation were seen when USP19 was silenced in PC-3 cells (Fig. S2). Therefore, the USP19 modulation of prostate cancer cell growth occurs through a regulation of cell cycle progression from G0/G1 to S phase and does not affect the rate of apoptotic cell death.

### Depletion of USP19 results in accumulation of p27^Kip1^

**Figure 3:**
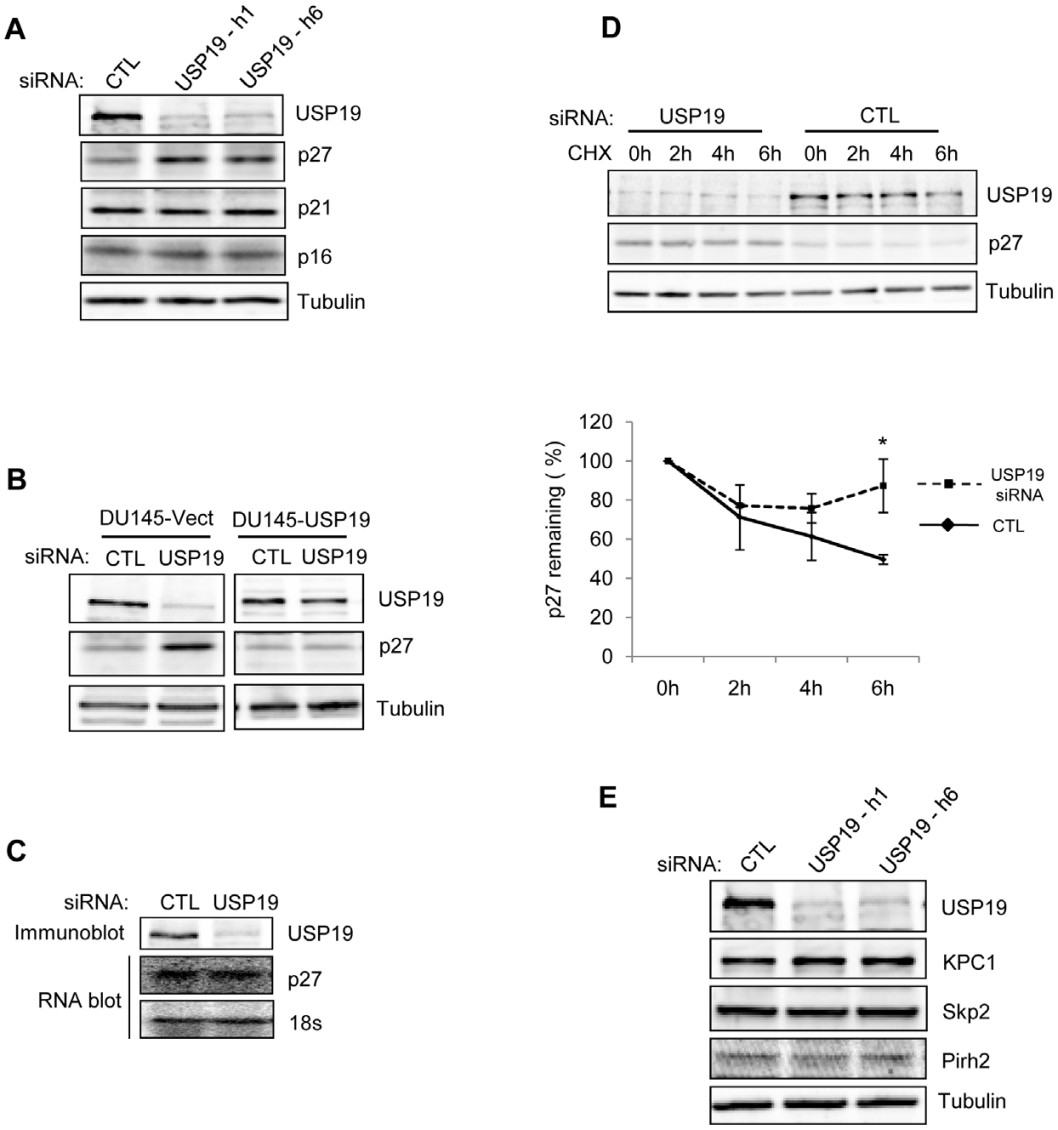
Depletion of USP19 results in accumulation of p27^Kip1^ in DU145 cells. (**A**) p27^Kip1^ but not p21 or p16 accumulates in USP19-depleted DU145 cells. Equal amounts of protein from lysates of cells transfected with control or USP19 siRNA oligonucleotides (as described in Fig. 1A) were analyzed by immunoblotting with the indicated antibodies. (**B**) Overexpression of USP19 in DU145 cells prevents USP19 siRNA induced increase of p27^Kip1^. Protein from lysates of cells transfected with control or USP19 siRNA oligonucleotides (as described in Fig. 1B) were analyzed by immunoblotting with the indicated antibodies. Note that for the USP19 immunoblots, 300 µg of DU145-Vect protein and 20 µg of DU145-USP19 protein were used. (**C**) Silencing of USP19 does not increase p27^Kip1^ levels by increasing mRNA levels. Equal amounts of protein and RNA isolated from lysates of DU145 cells transfected with USP19 siRNA oligonucleotide h1 or control oligonucleotide (CTL) (as described in Fig. 1A) were analyzed by Western (top) and Northern (bottom) blots, respectively. (**D**) Silencing of USP19 stabilizes p27^Kip1^. DU145 cells were transfected with USP19 siRNA oligonucleotide h1 or control oligonucleotide (CTL). Forty-eight hours later, they were incubated with cycloheximide (CHX) to inhibit protein synthesis. At the indicated times, cells were lysed and proteins were analyzed by immunoblotting with the indicated antibodies. Shown are representative immunoblots and quantitation of p27^Kip1^ levels (means ± SE) from triplicate samples. *, rate of degradation of p27^Kip1^ was significantly inhibited in USP19 depleted DU145 cells (*P*<0.05) (two-way analysis of variance). (**E**) Levels of ubiquitin protein ligases, KPC1, Skp2 and Pirh2 are not affected upon USP19 depletion in DU145 cells. Equal amounts of protein from lysates (as in Fig. 1A) were analyzed by immunoblotting with the indicated antibodies. Quantification was performed on triplicate samples and did not reveal any significant differences in the levels of the ligases.

Since depletion of USP19 delayed G0/G1 to S progression, we explored possible mechanisms by assessing the steady-state levels of CKIs that inhibit G0/G1-S transition. USP19 siRNA, but not control siRNA led to an accumulation of p27^Kip1^, but not p16 or p21 as exemplified in the DU145 model (Fig. 3A). To confirm that this increased p27^Kip1^ was specific to USP19 depletion, USP19 siRNA was transfected in the stable DU145 cell line expressing rat USP19. In agreement with above, depletion of USP19 in DU145 cells expressing empty vector led to an induction of p27^Kip1^. However, in cells overexpressing rat USP19, the same siRNA treatment was unable to upregulate p27^Kip1^ (Fig. 3B), confirming that the upregulation of p27^Kip1^ is a specific response to USP19 depletion. This increased p27^Kip1^ induced by USP19 depletion did not appear to be due to increased gene transcription, as Northern blot analysis revealed that p27^Kip1^ mRNA levels did not change (Fig. 3C). These data suggest an involvement of USP19 in regulating the turnover of p27^Kip1^. To test this possibility, the rate of disappearance of p27^Kip1^ was measured in DU145 cells with or without USP19 depletion following inhibition of protein synthesis with cycloheximide. Indeed, in cells transfected with USP19 siRNA p27^Kip1^ levels remained relatively stable over time whereas the half-life of the protein was significantly lower in controls (Fig. 3D), implying that USP19 modulates the stability of p27^Kip1^. To determine whether the upregulation and stabilization of p27^Kip1^ in USP19 depleted DU145 cells was linked to changes in KPC1 expression as we previously reported [20] or the other E3s, Skp2 and Pirh2, also responsible for the ubiquitination of p27^Kip1^, we measured the levels of these proteins in response to USP19 depletion. Unexpectedly, we did not observe any changes in KPC1 (Fig. 3E), nor in Skp2 and Pirh2 levels (Fig. 3E). These observations indicate that USP19 can act through a KPC1 and also Skp2 and Pirh2 independent pathway(s) while still modulating cell growth and p27^Kip1^. Of interest the silencing of USP19 in PC-3 and 22RV1 cells, which also led to an inhibition of their proliferation resulted in a similar upregulation of p27^Kip1^ expression but also no changes in levels of the three ubiquitin ligases, KPC1, Skp2 or Pirh2 (Fig. S3). These results suggest that USP19 may lose its ability to regulate growth such as in the LNCaP cells or still exert this function through p27^Kip1^ as in a variety of other prostate cancer cell lines but this occurs through a pathway that bypasses the KPC1 ligase.

### Depletion of USP19 results in reduced rates of cell growth and accumulation of p27^Kip1^ in MCF10A breast epithelial cells but not in MCF7 and MDA-MB-231 breast carcinoma cells

**Figure 4:**
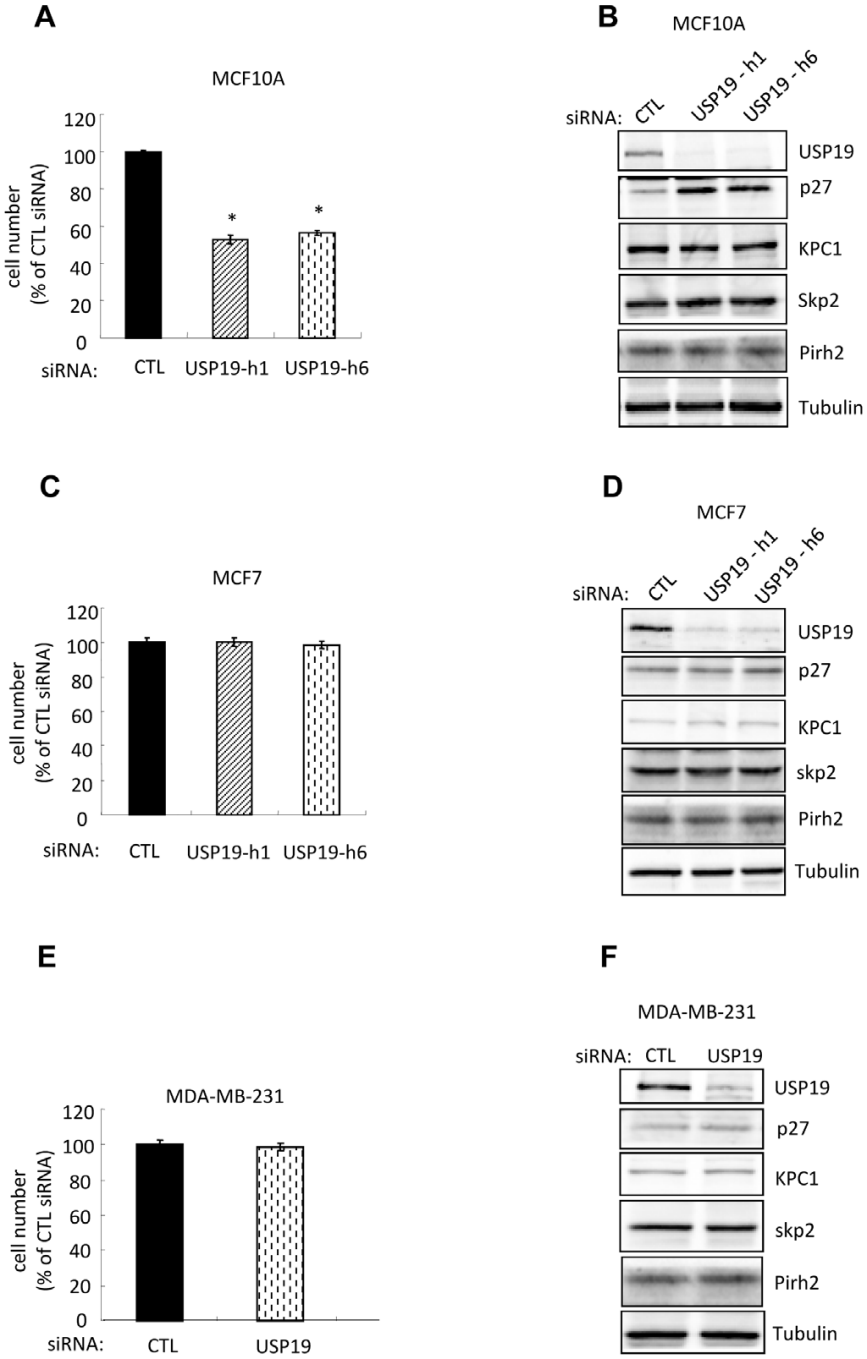
Depletion of USP19 inhibits growth in normal breast epithelial MCF10A but not in breast cancer MCF7 and MDA-MB-231 cells. (**A**) Breast epithelial MCF10A cells depleted of USP19 show reduced proliferation. MCF10A cells were transfected with USP19 siRNA oligonucleotides h1 or h6 or nonspecific control siRNA oligonucleotide (CTL). Forty-eight hours after transfection, cells were harvested and counted. Shown are means ± SE of triplicate samples. *, *P*<0.001 compared to CTL. (**B**) Depletion of USP19 in MCF10A cells results in a KPC1-independent accumulation of p27^Kip1^ in MCF10A cells. USP19 depleted MCF10A cells from experiment described in Fig. 4A were harvested. Equal amounts of protein from lysates were analyzed by immunoblotting with the indicated antibodies. (**C–F**) Depletion of USP19 does not lead to changes in cell growth or p27^Kip1^ levels in breast cancer MCF7 or MDA-MB-231 cells. Forty-eight hours after transfection with USP19 siRNA oligonucleotide h6 or nonspecific control siRNA oligonucleotide (CTL), cells (C and D) MCF7 and (E and F) MDA-MB-231 were harvested and counted (no significant difference in cell numbers between control and USP19 siRNAs). Equal amounts of protein from lysates were analyzed by immunoblotting with the indicated antibodies.

We next tested the role of USP19 in breast normal cells by depletion of USP19 in the immortalized MCF10A epithelial cells. This strategy resulted once more in ∼50% inhibition of cell growth (Fig. 4A) and elevation of p27^Kip1^ (Fig. 4B). Again levels of the E3 ligases, KPC1, Skp2 and Pirh2 did not change (Fig. 4B). Therefore, the upregulation of p27^Kip1^ in USP19 depleted MCF10A cells appeared to be independent of KPC1, Skp2, or Pirh2 as was seen in most of the prostate cancer cell lines.

We next asked whether the regulatory role of USP19 in breast normal epithelial cell growth applied in human breast cancer using the MCF7 and MDA-MB-231 carcinoma cell lines. Surprisingly, no changes in cell growth and in levels of p27^Kip1^ and the E3 ligases, KPC1 or Skp2 or Pirh2 were observed in either cell line upon USP19 depletion (Fig. 4C–F). Thus it appears that the ability of USP19 to regulate cell growth and p27^Kip1^ levels is lost in these breast carcinoma cells, similar to the situation noticed above in the LNCaP cells.

### Role of Ras in the ability of USP19 to regulate cell growth through p27^Kip1^ and KPC1

**Figure 5:**
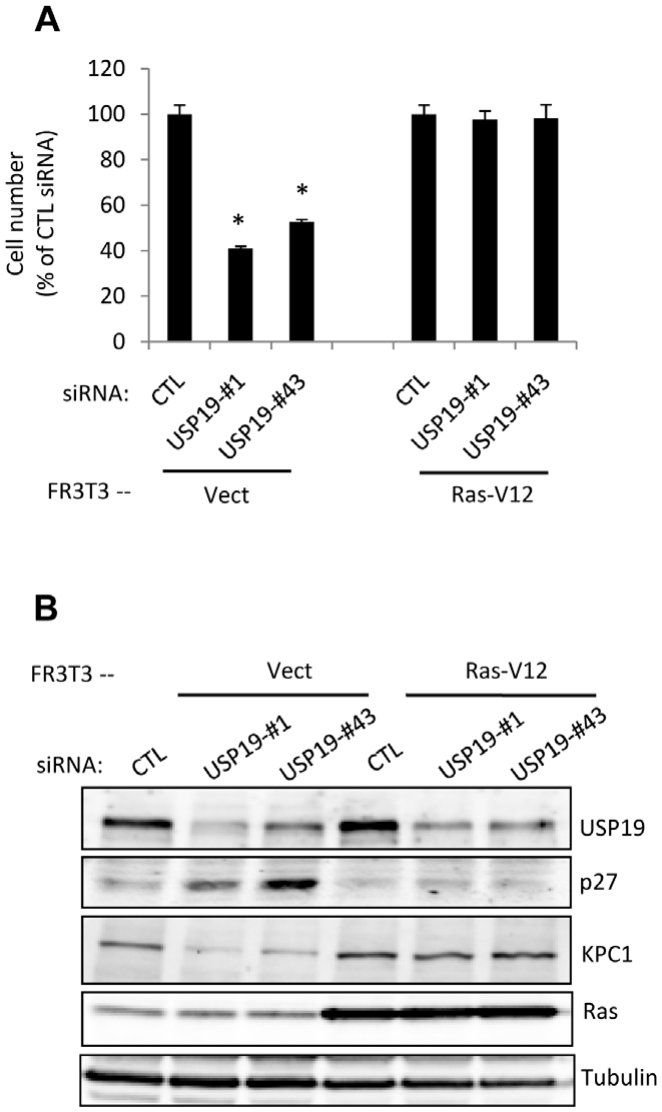
The ability of depletion of USP19 to regulate cell growth and p27^Kip1^ is lost in FR3T3 fibroblasts transformed by constitutively active Ras. (**A**) Rat FR3T3 fibroblasts expressing an activated Ras oncogene (Ras-V12) or empty vector were transfected with USP19 siRNA oligonucleotides #1, #43, or nonspecific control siRNA oligonucleotide (CTL). Forty-eight hours after transfection, cells were harvested and counted. Shown are means ± SE of triplicate samples. *, *P*<0.001 compared to CTL. (**B**) USP19 depleted FR3T3-vect or FR3T3-Ras cells from experiment described in Fig. 5A were harvested. Equal amounts of protein from lysates were analyzed by immunoblotting with the indicated antibodies.

The striking differences observed between the USP19 growth regulatory patterns in human breast cells, normal *vs* carcinoma, and prostate cancer cell lines as well as rat fibroblasts raised the question of whether the cell growth response to USP19 depletion may somehow be associated to oncogenic cell transformation. To test this possibility and further dissect underlying mechanisms, FR3T3 cells were transduced stably with a retrovirus expressing an activated Ras oncogene (Ras-V12) or an empty vector. The effects upon USP19 depletion were analyzed and compared to cells transfected with control oligonucleotide. As shown before, depletion of USP19 in control FR3T3 cells expressing empty vector resulted in growth inhibition (Fig. 5A) with a corresponding upregulation of p27^Kip1^ and reduction in KPC1 levels (Fig. 5B). However, in Ras transformed FR3T3 cells, depletion of USP19 no longer led to any of these changes (Fig. 5A and B). Therefore, the ability of USP19 to control p27^Kip1^ *via* KPC1 and thereby regulate cell growth may be abolished when cells are transformed *via* the Ras oncogene. Altogether, this strongly suggests that constitutive activation of the Ras signalling pathway may figure among mechanisms explaining altered USP19 function and p27^Kip1^ levels in cancer cells.

## Discussion

The present investigation explored, for the first time, the ability of USP19 to regulate growth in diverse cell models, with emphasis on human prostate and breast cell lines. The rationale was to determine if the novel mechanism that we established in rodent fibroblasts applies broadly or is restricted to specific cell systems. Our findings distinguished three distinct patterns of USP19 action (Table S1). In the first one, observed in fibroblasts (Fig. 5) and characterized in detail previously [20], USP19 stabilizes the KPC1 ligase and thereby enhances the ubiquitination of p27^Kip1^ in G1 and promotes cell proliferation.

In the second pattern, demonstrated in three prostate cancer cell lines (DU145, PC-3, 22RV1) and in the immortalized breast normal epithelial cell line, USP19 conserved its ability to positively regulate cell growth through stimulation of progression through G1 into S phase and enhanced the rate of degradation of p27^Kip1^ (Figs. 1, 2, 4A). However, in contrast to the situation depicted in fibroblasts, USP19 effects were not mediated through levels of the KPC1 ligase for p27^Kip1^. The underlying mechanism remains unclear but appears unrelated to levels of Skp2 or Pirh2, other ligases for p27^Kip1^ (Fig. 3E). There is a report that the RING finger ligase Ro52 binds with Skp2, Cul1, and Skp1 to mediate ubiquitination of p27^Kip1^ in HeLa cells [21]. In this complex, Ro52 appears to replace the RING finger protein Rbx1that is typically found in Cul1 based cullin ring ligases and therefore appears to support ubiquitination of p27^Kip1^ only indirectly through Skp2. Nonetheless, we have measured Ro52 in DU145 cells with and without silencing of USP19 and also did not observe any differences in levels of this protein (data not shown). It is possible that USP19 may modulate yet other ligases for p27^Kip1^ that have not been uncovered. In addition, we cannot exclude that p27^Kip1^ is also targeted by a non-ubiquitin proteasome dependent or independent proteolytic mechanism whose activity is promoted by USP19. Although p27^Kip1^ mRNA levels remain unchanged upon USP19 depletion (Fig. 3C), it remains possible that p27^Kip1^ synthesis is regulated also at the level of translation and that USP19 modulates such translational efficiency of p27^Kip1^ mRNA. All of these possibilities indicate that USP19 has additional substrates that remain to be identified. Considering that the number of DUBs encoded in the genome is approximately one tenth the number of E3s, it is not surprising that a DUB may have multiple substrates and therefore act on multiple regulatory pathways in the cell. The net effects of these regulatory mechanisms, present in varying degrees in different cell types could lead to divergent outcomes. A recent large scale proteomic analysis of interacting proteins of deubiquitinating enzymes, including USP19, has been reported [22]. Many potential interacting proteins for USP19 were identified, but none appeared to be putative ubiquitin protein ligases or known modulators of G1 to S progression.

A limitation of this large scale proteomic study was that it was carried out in human embryonic kidney HEK293 cells. As demonstrated by our findings, the effects of USP19 and its mechanisms of action appear to be cell type dependent. Indeed, silencing of USP19 did not alter growth rates of HEK293 cells (unpublished data) and so the target of USP19 that mediates its effects on growth may not be present in these cells. The identification of USP19 associated molecules and of potentially novel substrates clearly stem as important questions arising from our study. However, the search for such proteins, destabilized and/or more ubiquitinated upon USP19 depletion, needs precisely to be conducted in a cell specific context.

The third observed pattern was a complete loss of the ability of USP19 to regulate cell growth and p27^Kip1^ levels. This was seen in the prostate cancer LNCaP model, the breast cancer MCF7 and MDA-MB-231 models (Fig. 4C–F; Fig S1B), and in fibroblasts once transformed by the Ras oncogene (Fig. 5). Interestingly, although USP19 could regulate cell growth in MCF10A breast epithelial cells, this regulation was lost in breast cancer MCF7 and MDA-MB-231 cells. This suggested that some aspect of oncogenic transformation can disrupt USP19 mediated regulation. Indeed, we confirmed this hypothesis by demonstrating that the regulatory role of USP19 in fibroblasts can be completely disrupted by transforming the cells with expression of constitutively active Ras. This aspect of transformation may also be present in LNCaP cells, but not in the prostate cancer cell lines that were still inhibitable by USP19 depletion.

To date there are very few examples of deubiquitinating enzymes that have been implicated in tumorigenesis. Mutations in the CYLD deubiquitinating enzyme leads to familial cylindromatosis [23] and overexpression of USP2 has been reported to increase prostate cancer cell proliferation through the stabilization of fatty acid synthase [24]. Very recently, USP17 has been identified to be highly expressed in primary lung, colon, esophagus, and cervical cancers and also modulates the stability of p27^Kip1^ in G1 [25]. It appears from expression profiling studies that USP19 mRNA transcripts do not increase in breast tumors (Hallett, M and Park M, unpublished data) nor in prostate cancers [26] compared to their normal adjacent tissues. Along this line, our attempts to assess levels of USP19 in human prostate cancers by immunohistochemistry were technically not conclusive, due to low expression levels in both malignant and normal prostate tissues. Although we have no evidence yet of increased expression of USP19 in cancer, it is worth noting that most prostate cancer cell lines, in particular androgen independent ones, were still sensitive to loss of USP19. This suggests that pharmacologic inhibition of USP19 activity may be a useful novel approach for treatment of prostate cancer, including those tumors that have become unresponsive to androgen deprivation and often lead to death.

Collectively, our findings illustrate that depending on the cellular context, USP19 can regulate growth in a KPC1 dependent manner or in a KPC1 independent manner, or have no effect on cell growth. Certain modifications that can occur upon oncogenic transformation may be responsible for these differences. These alterations could include changes in the substrates of USP19. To help identify these substrates, it would be of interest to compare the pattern of proteins that are destabilized or more ubiquitinated upon USP19 silencing in cells whose growth is sensitive to USP19 depletion with the pattern of such proteins found in cells whose growth is insensitive to USP19 depletion.

## Supporting Information

Figure S1 **Depletion of USP19 results in reduced rates of cell growth of the androgen- sensitive 22RV1 but not of LNCaP cell lines.** (**A**) 22RV1 cells depleted of USP19 show reduced proliferation. Forty-eight hours after transfection with USP19 siRNA oligonucleotide h1 or nonspecific control siRNA oligonucleotide (CTL), 22RV1 cells were harvested and counted. Shown are means ± SE of triplicate samples. *, *P*<0.001 compared to CTL. (**B**) Depletion of USP19 did not change growth rates of LNCaP cells. Forty-eight hours after transfection with USP19 siRNA oligonucleotide h1 or h6 or nonspecific control siRNA oligonucleotide (CTL), LNCaP cells were harvested and counted. Shown are means ± standard error of triplicate samples (no significant difference from results for control siRNA).
(TIF)

Figure S2 **Depletion of USP19 in PC-3 cells results in defects in cell cycle progression.** PC-3 cells depleted of USP19 show accumulation in G1 phase and decreased entry into S phase. Forty-eight hours after transfection with USP19 siRNA oligonucleotide h1 or control oligonucleotide (CTL), PC-3 cells were either harvested and subjected to FACS analysis (top panel) or incubated with BrdU (10 µM) for 55 min and analyzed by FACS to quantify S phase cells (identified as BrdU positive cells and identified by circles) (lower panel). Shown are representative profiles. *, *P*<0.001 compared to CTL.
(TIF)

Figure S3 **Depletion of USP19 results in accumulation of p27^Kip1^ in PC-3 and 22RV1 cells.** Silencing of USP19 in PC-3 and 22RV1 cells increases p27^Kip1^ levels, but does not change the levels of KPC1, Skp2 and Pirh2. Equal amounts of protein from lysates were analyzed by immunoblotting with the indicated antibodies.
(TIF)

Table S1 **Three distinct patterns of effects of silencing of USP19 on cell growth, KPC1 ligase and p27^Kip1^.**
(TIF)

## References

[1] Morgan DO (1995). Principles of CDK regulation.. Nature.

[2] Malumbres M, Barbacid M (2005). Mammalian cyclin-dependent kinases.. Trends Biochem Sci.

[3] Sherr CJ, Roberts JM (1999). CDK inhibitors: positive and negative regulators of G1-phase progression.. Genes Dev.

[4] Chu IM, Hengst L, Slingerland JM (2008). The Cdk inhibitor p27 in human cancer: prognostic potential and relevance to anticancer therapy.. Nat Rev Cancer.

[5] Fero ML, Rivkin M, Tasch M, Porter P, Carow CE (1996). A syndrome of multiorgan hyperplasia with features of gigantism, tumorigenesis, and female sterility in p27(Kip1)-deficient mice.. Cell.

[6] Kiyokawa H, Kineman RD, Manova-Todorova KO, Soares VC, Hoffman ES (1996). Enhanced growth of mice lacking the cyclin-dependent kinase inhibitor function of p27(Kip1).. Cell.

[7] Nakayama K, Ishida N, Shirane M, Inomata A, Inoue T (1996). Mice lacking p27(Kip1) display increased body size, multiple organ hyperplasia, retinal dysplasia, and pituitary tumors.. Cell.

[8] Guan X, Wang Y, Xie R, Chen L, Bai J (2009). p27 as a prognostic factor in breast cancer: a systematic review and meta-analysis.. J Cell Mol Med.

[9] Pagano M, Tam SW, Theodoras AM, Beer-Romero P, Del Sal G (1995). Role of the ubiquitin-proteasome pathway in regulating abundance of the cyclin-dependent kinase inhibitor p27.. Science.

[10] Pickart CM (2001). Mechanisms underlying ubiquitination.. Annu Rev Biochem.

[11] Kamura T, Hara T, Matsumoto M, Ishida N, Okumura F (2004). Cytoplasmic ubiquitin ligase KPC regulates proteolysis of p27(Kip1) at G1 phase.. Nat Cell Biol.

[12] Hara T, Kamura T, Kotoshiba S, Takahashi H, Fujiwara K (2005). Role of the UBL-UBA protein KPC2 in degradation of p27 at G1 phase of the cell cycle.. Mol Cell Biol.

[13] Kotoshiba S, Kamura T, Hara T, Ishida N, Nakayama KI (2005). Molecular dissection of the interaction between p27 and Kip1 ubiquitylation-promoting complex, the ubiquitin ligase that regulates proteolysis of p27 in G1 phase.. J Biol Chem.

[14] Carrano AC, Eytan E, Hershko A, Pagano M (1999). SKP2 is required for ubiquitin-mediated degradation of the CDK inhibitor p27.. Nat Cell Biol.

[15] Tsvetkov LM, Yeh KH, Lee SJ, Sun H, Zhang H (1999). p27(Kip1) ubiquitination and degradation is regulated by the SCF(Skp2) complex through phosphorylated Thr187 in p27.. Curr Biol.

[16] Hattori T, Isobe T, Abe K, Kikuchi H, Kitagawa K (2007). Pirh2 promotes ubiquitin-dependent degradation of the cyclin-dependent kinase inhibitor p27Kip1.. Cancer Res.

[17] Shimada M, Kitagawa K, Dobashi Y, Isobe T, Hattori T (2009). High expression of Pirh2, an E3 ligase for p27, is associated with low expression of p27 and poor prognosis in head and neck cancers.. Cancer Sci.

[18] Nijman SM, Luna-Vargas MP, Velds A, Brummelkamp TR, Dirac AM (2005). A genomic and functional inventory of deubiquitinating enzymes.. Cell.

[19] Reyes-Turcu FE, Wilkinson KD (2009). Polyubiquitin binding and disassembly by deubiquitinating enzymes.. Chem Rev.

[20] Lu Y, Adegoke OA, Nepveu A, Nakayama KI, Bedard N (2009). USP19 deubiquitinating enzyme supports cell proliferation by stabilizing KPC1, a ubiquitin ligase for p27Kip1.. Mol Cell Biol.

[21] Sabile A, Meyer AM, Wirbelauer C, Hess D, Kogel U (2006). Regulation of p27 degradation and S-phase progression by Ro52 RING finger protein.. Mol Cell Biol.

[22] Sowa ME, Bennett EJ, Gygi SP, Harper JW (2009). Defining the human deubiquitinating enzyme interaction landscape.. Cell.

[23] Bignell GR, Warren W, Seal S, Takahashi M, Rapley E (2000). Identification of the familial cylindromatosis tumour-suppressor gene.. Nat Genet.

[24] Priolo C, Tang D, Brahamandan M, Benassi B, Sicinska E (2006). The isopeptidase USP2a protects human prostate cancer from apoptosis.. Cancer Res.

[25] McFarlane C, Kelvin AA, de la Vega M, Govender U, Scott CJ The deubiquitinating enzyme USP17 is highly expressed in tumor biopsies, is cell cycle regulated, and is required for G1-S progression.. Cancer Res.

[26] Lapointe J, Li C, Higgins JP, van de Rijn M, Bair E (2004). Gene expression profiling identifies clinically relevant subtypes of prostate cancer.. Proc Natl Acad Sci U S A.

