# Supplementary figures and images for: Identification of Distinctive Patterns of USP19-Mediated Growth Regulation in Normal and Malignant Cells
Source: PLoS One. 2011 Jan 17;6(1):e15936. doi: 10.1371/journal.pone.0015936 (PMC3022023; doi:10.1371/journal.pone.0015936)

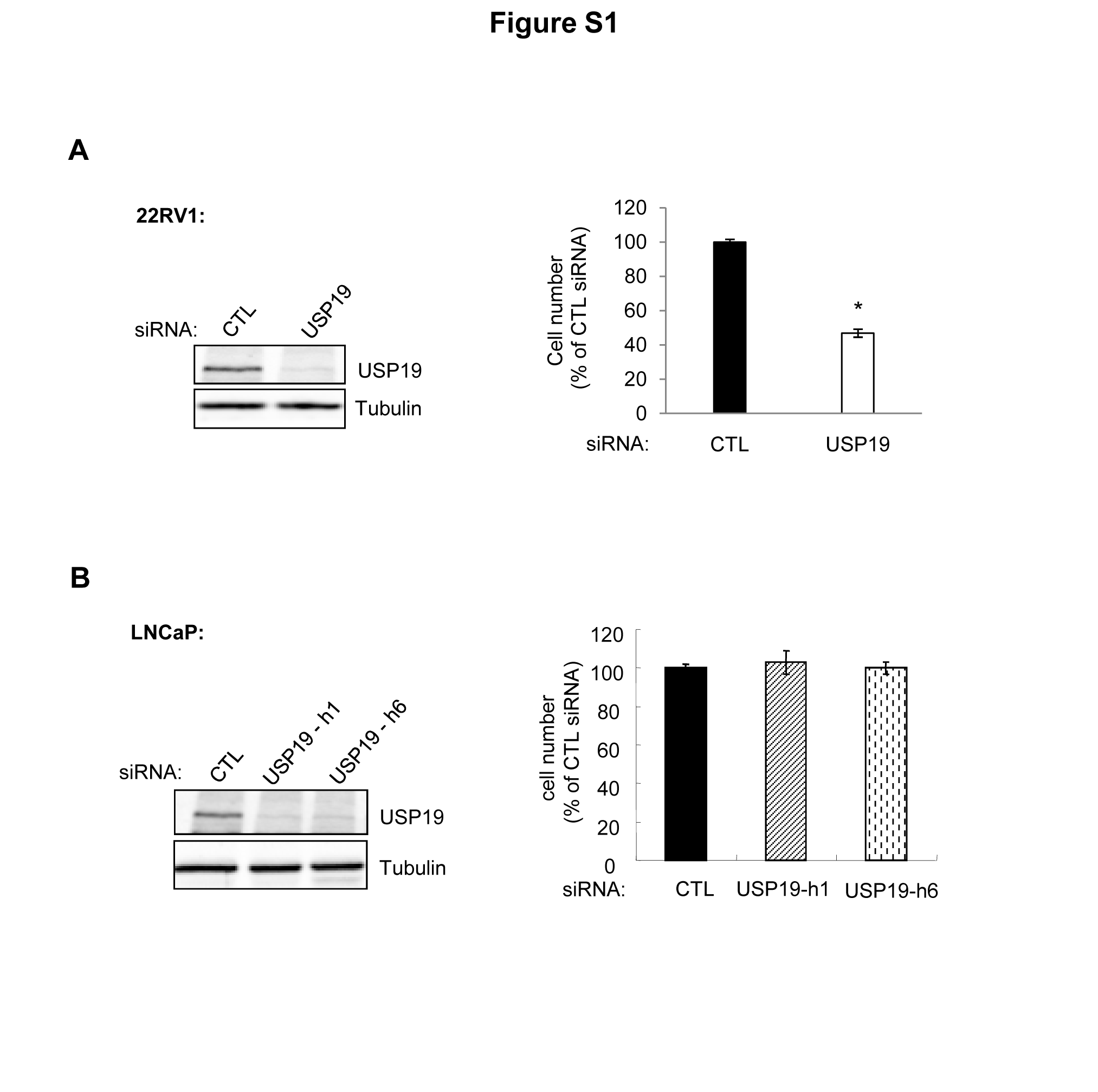

Supplement: Figure S1 — Depletion of USP19 results in reduced rates of cell growth of the androgen- sensitive 22RV1 but not of LNCaP cell lines. (A) 22RV1 cells depleted of USP19 show reduced proliferation. Forty-eight hours after transfection with USP19 siRNA oligonucleotide h1 or nonspecific control siRNA oligonucleotide (CTL), 22RV1 cells were harvested and counted. Shown are means ± SE of triplicate samples. *, P<0.001 compared to CTL. (B) Depletion of USP19 did not change growth rates of LNCaP cells. Forty-eight hours after transfection with USP19 siRNA oligonucleotide h1 or h6 or nonspecific control siRNA oligonucleotide (CTL), LNCaP cells were harvested and counted. Shown are means ± standard error of triplicate samples (no significant difference from results for control siRNA). (TIF) [file pone.0015936.s001.tif]

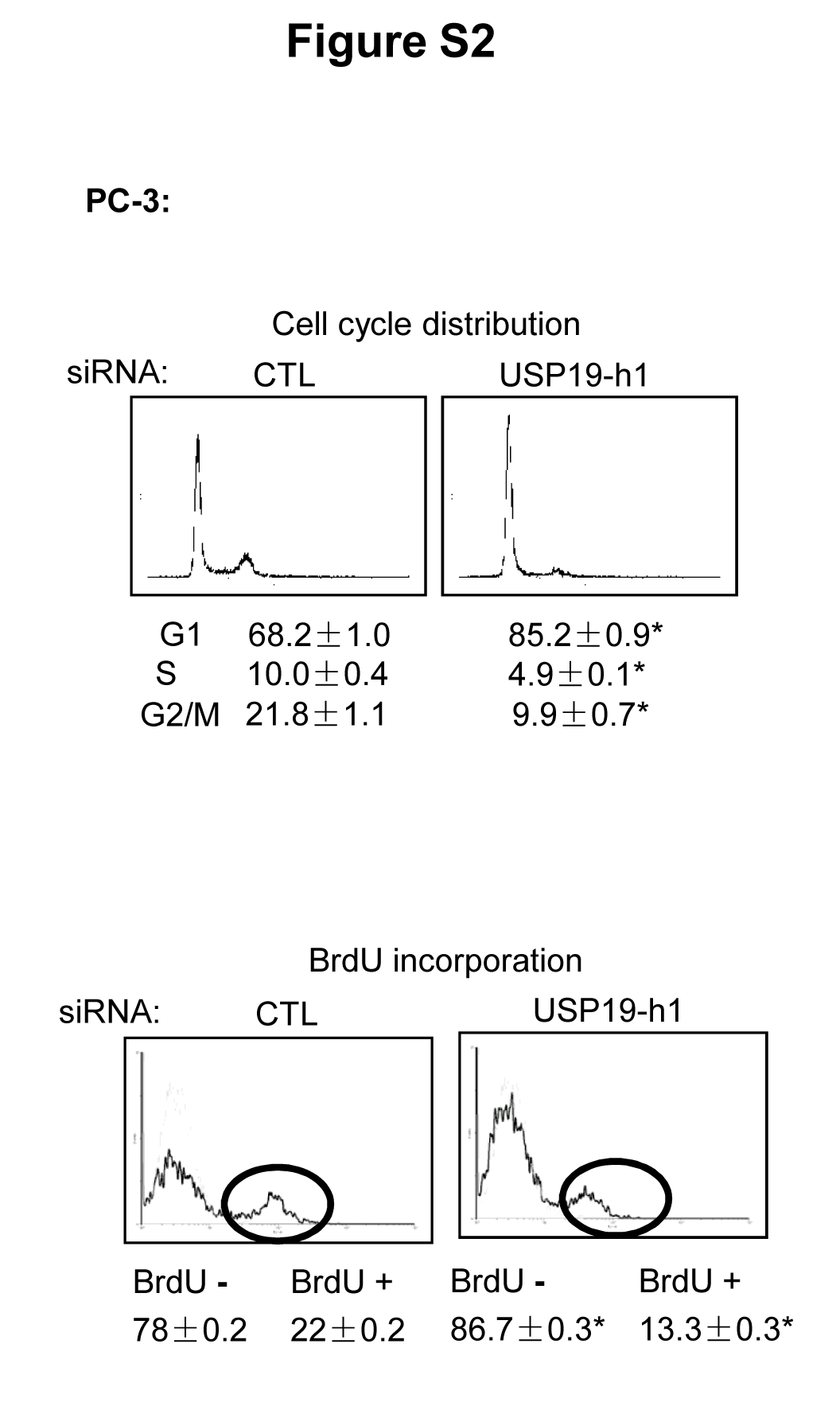

Supplement: Figure S2 — Depletion of USP19 in PC-3 cells results in defects in cell cycle progression. PC-3 cells depleted of USP19 show accumulation in G1 phase and decreased entry into S phase. Forty-eight hours after transfection with USP19 siRNA oligonucleotide h1 or control oligonucleotide (CTL), PC-3 cells were either harvested and subjected to FACS analysis (top panel) or incubated with BrdU (10 µM) for 55 min and analyzed by FACS to quantify S phase cells (identified as BrdU positive cells and identified by circles) (lower panel). Shown are representative profiles. *, P<0.001 compared to CTL. (TIF) [file pone.0015936.s002.tif]

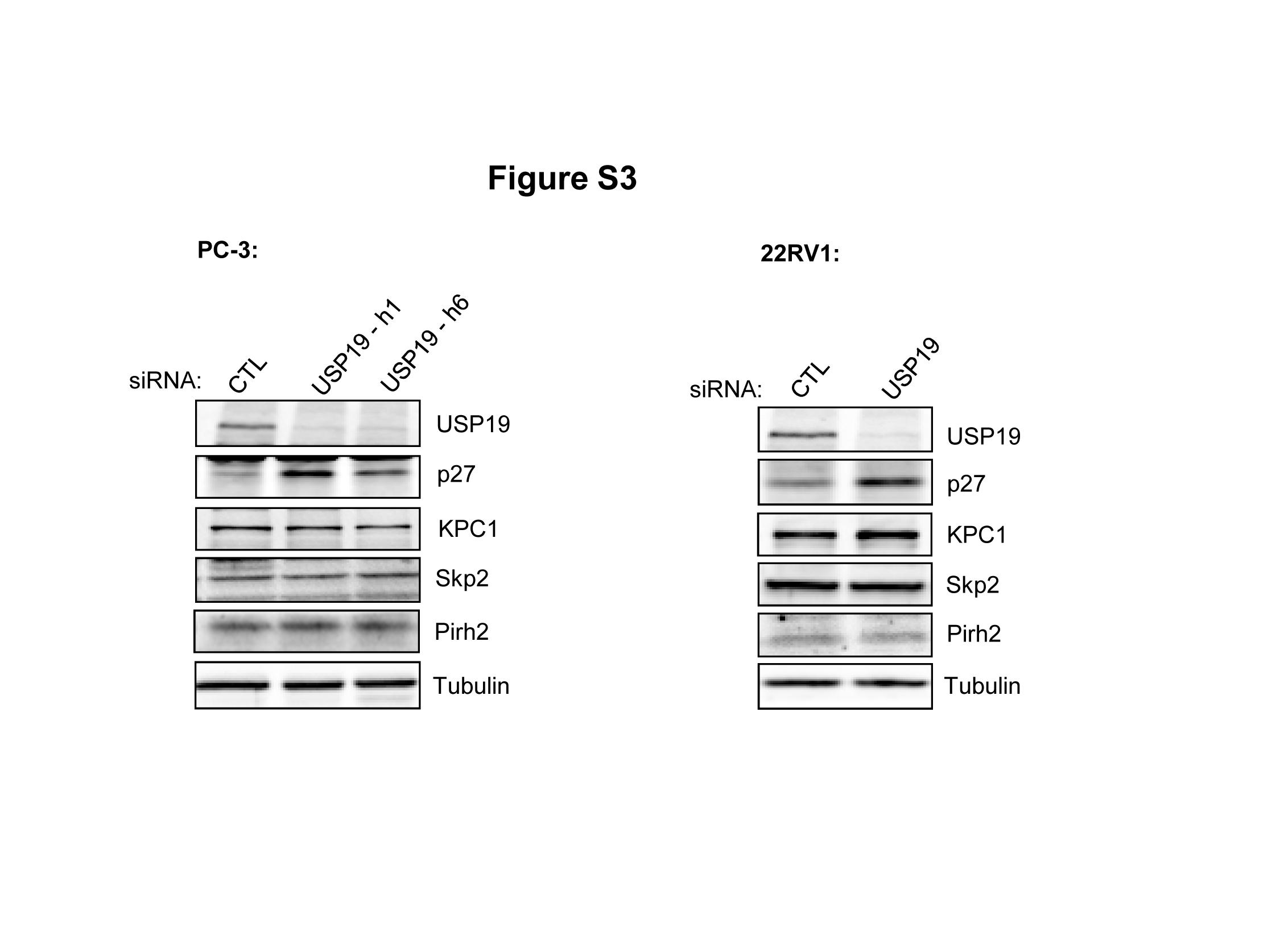

Supplement: Figure S3 — Depletion of USP19 results in accumulation of p27Kip1 in PC-3 and 22RV1 cells. Silencing of USP19 in PC-3 and 22RV1 cells increases p27Kip1 levels, but does not change the levels of KPC1, Skp2 and Pirh2. Equal amounts of protein from lysates were analyzed by immunoblotting with the indicated antibodies. (TIF) [file pone.0015936.s003.tif]

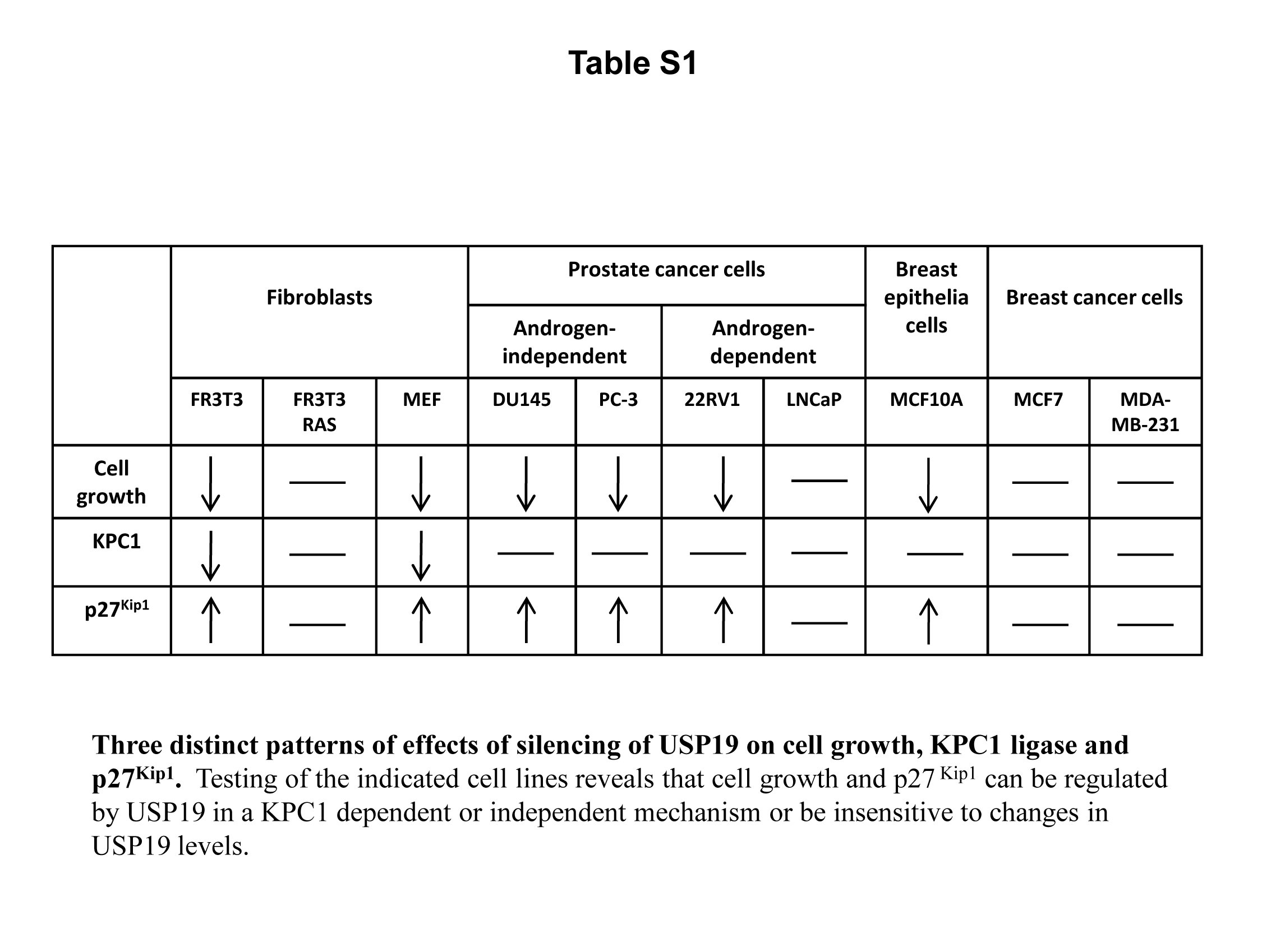

Supplement: Table S1 — Three distinct patterns of effects of silencing of USP19 on cell growth, KPC1 ligase and p27Kip1. (TIF) [file pone.0015936.s004.tif]
